# Co-Developing Patient-Centered Information: A Focus Group Study of Asthma Patients’ Preferences and Attitudes towards New Medical Treatment Guidelines

**DOI:** 10.3390/ph16030456

**Published:** 2023-03-17

**Authors:** Sara Sommer Holst, Charlotte Vermehren

**Affiliations:** 1 Department of Clinical Pharmacology, Copenhagen University Hospital Bispebjerg, DK-2400 Copenhagen, Denmark; 2 Faculty of Health and Medical Sciences, University of Copenhagen, DK-2200 Copenhagen, Denmark

**Keywords:** asthma, pharmacological asthma treatment, treatment change, patient information, development, patient involvement, focus group, preference, attitude

## Abstract

Studies have suggested patient involvement as an important factor when seeking to improve patient-centered information. The objective of this study was to explore asthma patients’ preferences regarding information when co-developing patient-centered information and how they evaluate the material as a supportive initiative when they are deciding whether to switch to the new MART approach. The study was performed as a case study involving qualitative semi-structured focus group interviews inspired by the theoretical framework for supporting patient involvement in research. Two focus group interviews were held, with a total of nine interviewees. Three main interview themes were found: the identification of important topics about the new MART approach, feedback on the design and the preferred implementation of written patient-centered information. The asthma patients preferred written patient-centered material to be short and to be presented briefly at the local community pharmacy, and then discussed more thoroughly with their general practitioner (GP) at a consultation. In conclusion, this study identified asthma patients’ preferences when co-developing written patient-centered information and how the patients favored the material to be implemented as a support to them in their decision on whether to change asthma treatment.

## 1. Introduction

Asthma has a disease prevalence of 7–11% in Denmark and is considered to be a common disease associated with airway inflammation [1]. The aim of the pharmacological treatment of asthma is to achieve as normal lung function as possible, few as possible symptoms and reduce the risk of exacerbations [2]. Therefore, it is standard that asthma patients will receive both maintenance and reliever therapy, depending on asthma severity.

In 2019, the Global Initiative for Asthma (GINA) published new guidelines for the pharmacological treatment of adult asthma patients, i.e., the recommended line of treatment for all asthma patients, regardless of their asthma severity, was to receive inhaled corticosteroids (ICS) in combination with a long-acting β_2_-agonist (LABA) as reliever therapy [3]. This combination treatment could also be used as a maintenance therapy based on the severity of the asthma condition, i.e., maintenance and reliever therapy (MART) [3]. These recommendations have not been changed markedly in the newest GINA guideline from 2022 [2]. GINA also indorses the use of formoterol as the most favorable LABA due to its long duration of action and rapid onset of action [2]. Furthermore, GINA highlighted that short-acting β_2_-agonist (SABA) should not be used as monotherapy, as evidence showed that SABA-only treatment increased the risk of severe exacerbations, and that adding ICS reduced the risk significantly [2].

A summary of the GINA Guidelines from 2018 compared with the GINA guidelines from 2022 is presented in Table 1.

The new MART approach reduces exacerbations 40–50% compared to a treatment where ICS-formoterol is used as a preventive treatment alongside a short-acting β_2_-agonist (SABA) used as needed [2]. With the MART approach, ICS is inhaled daily while providing additional doses of ICS as soon as symptoms appear, which reduces the risk of severe exacerbations and hospitalization [4,5].

The new MART approach is associated with many advantages, as mentioned above. However, it is known that asthma patients’ adherence is poor, ranging from 30 to 70%, and it is associated with reduced health care outcomes such as ineffectively controlled asthma and an increased risk of flare-ups [6,7,8,9]. Consequently, an unceasing focus is to improve the adherence of asthma patients, especially when implementing new treatment guidelines such as the MART approach.

Many studies have explored ways to improve adherence in asthma patients [10,11,12,13,14]. One example of an intervention that could improve asthma management and enhance the recognition of symptoms is education focusing on asthma self-management, leading to reduced emergency department re-attendance [12].

In addition to an increasing focus on improving adherence in asthma patients’ medical treatment, a new interest is also the engagement of patients in the developing phases of patient-centered initiatives, as some argue that patients have a right to give input to research on their condition [15]. Another argument for improving patient engagement in patient-centered initiatives is that patient involvement improves the efficiency and value of research via, e.g., increasing its relevance to patients by bringing a real-world and lived-experience perspective [16].

A comprehensive systematic review by Greenhalgh et al. from 2019 identified and synthesized frameworks for supporting patient involvement in research, where they considered whether and how these were used and applied design principles to improve usability [17]. The review found five main categories in which patient involvement was applied, especially the third study-focused category, designed to maximize recruitment and retention to clinical trials to improve the quality and efficiency of research, which have been investigated thoroughly in the past. The review found 19 different frameworks, where the clinical trials used a more-or-less linear model with proposals for patient involvement into every stage—from identifying and prioritizing research, design planning, the development of grant proposals, undertaking/managing the research, analyzing and interpreting research results to the dissemination, implementation and evaluation of the study findings [17].

A qualitative focus group interview study by Armstrong et al. from 2017 investigated patient preferences for an introduction of patient representatives in health care professional panels developing clinical guidelines. The study found that the patients were very interested in participating in the development of clinical guidelines [18]. Another qualitative focus group interview study by Gierisch et al. from 2019 found that patients could help to define and prioritize areas of interest for the research, translate findings into layman’s language, and identify clinically relevant outcomes in research [19]. A review by van Beusekom et al. from 2018 investigated the extent of patient involvement in the design and evaluation of pharmaceutical pictograms and endeavored to find evidence on whether involvement in the design process could increase the success of pictogram-enhanced drug information [20]. This review showed that involving patients in the design process helped to increase the likelihood that resulting pictograms were well-understood, and aided the understanding and recall of drug information [20]. Newer studies have also utilized literature reviews to summarize knowledge about patients’ need for information in specific chronic disease situations [21,22]. 

We have previously investigated asthma patients’ attitudes towards changing medical asthma treatment according to new treatment guidelines to identify factors that may be crucial for a successful, i.e., adherent, switch to the new MART approach [23]. The study showed that an increased focus on improving patient involvement during consultations with their general practitioner (GP) was a key initiative that may influence successful treatment change in asthma patients. It is also suggested that a written patient-centered information leaflet could be used to improve health care professional and patient dialogue both at general practice level and at local community pharmacies [23].

Even though many studies have suggested patient education, patient information and better patient and healthcare professional dialogue as means to improve drug adherence [10,11,12,13], and other studies have pointed out patient involvement as an important factor when seeking to improve patient-centered initiatives and information [18,19,20], it has not to our knowledge previously been investigated how a specific group of patients suffering from chronic diseases such as asthma could contribute to the development of written patient information in relation to new treatment guidelines. Studies investigating patient involvement in, e.g., risk information in surgical care have previously focused on the health care professionals’ opinions about the information or on researchers using validated scoring tools to evaluate patient information in relation to maternity care [24,25,26]. Therefore, the objective of this study was to elaborate on our previous study [23] by exploring asthma patients’ preferences regarding information when co-developing patient-centered information, and how they evaluate the material as a supportive initiative when they are deciding whether to switch to the new MART approach. The study was performed with a specific focus on:

1. Asthma patients’ preferences for the design and content of written patient-centered information.
2. Asthma patients’ attitude towards how the written patient-centered information could support them in decision-making regarding their pharmacological treatment change.

Hence, the ambition of this study was to present patient-developed key factors influencing the willingness to adhere to new treatment guidelines. This knowledge can potentially be transferred to other medical settings.

## 2. Results

Both focus groups lasted three hours, including a dinner break of 30 min. The first focus group interview consisted of five asthma patients and the second consisted of four asthma patients. A summary of the interviewees is presented in Table 2. 

Three themes and ten subthemes were derived from the analysis of the two focus groups. Interview themes, subthemes, and examples of quotes for the two focus group interviews are presented in Table 3.

### 2.1. Identification of Important Topics about the New MART Approach

The patients from the first and second focus group interviews were positive about changing their medical asthma treatment according to the new MART approach. They saw the new approach as good, as it adjusts the dose according to the variation in the severity of asthma and thus the patient receives only the necessary medication. However, all patients expressed concerns about the effectiveness of the new approach. The effectiveness of the new approach was important to the patients, and an improvement in the effectiveness was, by the patients, assessed as being a decisive motivation for changing their treatment. Furthermore, the patients agreed that they would like to have information about the effect and usage of the new MART approach compared to their regular asthma treatment, and that these were two important factors that should be well described in a written patient-centered information leaflet.

There was no consensus in the attitudes toward possible side effects of the new MART approach in the first and second focus group interview. Patients from the first focus group all agreed that it was important to obtain information about the side effects of the new approach, as too many side effects would deter them from changing treatment. However, patients from the second focus group interview thought that side effects were not the most important factor influencing their decision of whether to change to the new treatment, as they believed that they would experience the same side effects with the new approach as with the old one, and that these side effects were easy to handle. 

The patients from both focus group interviews all agreed that they would like practical information on how they should take the medication according to the new approach. The patients also agreed that both the price and availability of the new treatment would influence their attitude towards changing medication.

The patients from both focus group interviews expressed insecurities about having to assess their need for treatment themselves, and thereby assess when they should take their medication. The interviewees were unsure whether they would end up taking too much or too little medicine with the new MART approach, because it was designed to be used as needed instead of regularly daily. 

### 2.2. Feedback on the Design of Written Patient-Centered Information

The patients from both focus group interviews were generally very positive about a leaflet with patient information about the new MART approach. They saw several advantages in using the leaflet in a conversation with their GP regarding a discussion of a switch to the new approach. However, the patients emphasized that the leaflet should be kept short and precise.

Several of the patients from the first focus group interview thought that receiving information in writing in the form of a leaflet would help them to better remember the information they received from the GP. In addition, the patients believed that the leaflet could be used to increase their own involvement in the conversation with their GP when they had to discuss the possibility of changing their asthma treatment according to the new MART approach. Here, one of the interviewees argued, that patients would be able to use the leaflet to prepare before a consultation with their doctor, and the leaflet would provide basic knowledge so that the patient could make an informed decision.

The interviewees pointed out that the length of the information material must be as short as possible, as there is an abundance of information everywhere and the information in the leaflet could easily be overlooked. 

### 2.3. Implementation of Written Patient-Centered Information

The interviewees from both focus group interviews had different attitudes towards how written patient-centered information could be implemented in different settings. However, the patients from both focus group interviews agreed that they would prefer to receive brief information about the new MART approach from the local community pharmacy, and then have the longer discussion and decision about a possible change with their GP.

The patients believed that the GP was responsible for their medical treatment, which was why they wanted to talk to their GP about possible medical treatment changes. The patients from the first focus group interview generally trusted their GP a lot, while the patients from the second focus group interview were more critical. They believed that there was currently no proper discussion of the advantages and disadvantages of their medical treatment in the conversation with the GP. A few of the patients from the second interview also pointed out that GP consultations sometimes involved small talk instead of dealing with the actual problems, and that the doctors did not want to devote extra time to their patients for financial reasons.

The patients agreed that they did not want to discuss a possible change of their medical asthma treatment with the community pharmacy, but that it was alright if the pharmacy gave them brief information about the new MART approach and encouraged them to contact their GP.

Some of the patients believed that the pharmacy was a commercial entity that focused more on profit than on health professional advice, and that GPs had more authority than the pharmacy. In addition, it was also discussed whether or not the pharmacies would cross a line by informing patients about new medical treatment, which was normally the domain of the GP, according to the patients. It was also mentioned by a single patient that the pharmacy was too public a place to discuss something as private as one’s medical treatment.

Other patients were more divided, and saw both advantages and disadvantages of seeking information and advice at the local community pharmacy about the new MART approach. Here, it was pointed out by the patients that there was a big difference between the pharmacies. A few of the interviewees thought, for example, that trust in the pharmacy increased when you are used to coming there, which also was why they estimated that they would prefer to be informed about the new approach at a well-known pharmacy.

The patients from the second focus group interview were also asked how they would like to be presented the written patient information. The patients agreed that the leaflet should not just be handed out, but that it should be presented orally either at the pharmacy or used actively in the conversation with the GP before it made sense. A few of the interviewees believed that there would not be enough time to read the leaflet thoroughly in the doctor’s office. Therefore, they suggested that the leaflet could be handed out in the waiting room, so that you had time to read it before going to the GP.

## 3. Discussion

This study uniquely identified asthma patients’ preferences when co-developing written patient-centered information and assessing how the patients favored the material to be implemented as a support for them in their decision on whether to change asthma treatment. This knowledge may be contributory in understanding comparable situations in other pharmacological settings. It must be stressed that the present study is based on two focus group interviews with a total of nine interviewees and composed in a Danish setting. It is not our intent to generalize from this single case study, but rather to elucidate patient preferences in a co-development process of written patient-centered information.

The results from the focus group interviews showed that the asthma patients had clear preferences as to the design and content of a patient-centered information leaflet, i.e., they identified relevant topics and gave feedback on format.

The asthma patients clearly stated that information about the effect, side-effects and use of the new MART approach were most important to them in relation to decisions about treatment change and were key factors influencing their willingness to adhere to new treatment guidelines. A patient-centered information leaflet covering these important patient-reported topics may influence a more adherent asthma treatment. However, more research on this is needed.

The fact that the asthma patients were able to contribute successfully to the development of written information correlates with previous findings in a different setting, where van Beusekom et al. reviewed the literature about patient involvement in the design and evaluation of pictograms to support patient drug information [20]. The study found that involving end-users in the development of pharmaceutical pictograms helped to increase the likelihood that the resulting pictograms were well-understood and aided understanding and recall of drug information. They also stated that pictogram outcomes could be improved by involving participants in the design, but that patients were often only involved in the final evaluation of the pictogram and not the actual development [20]. Based on the results from the present study, it is clear that patient contributions to the development phase will benefit the information leaflet.

Furthermore, when the asthma patients gave feedback on the written patient-centered information, they emphasized that the written information should be kept short and precise. The patients agreed that written information should not just be handed out as a leaflet, but should be either presented orally at the local community pharmacy or used actively in the conversation with the GP so that it made sense.

The same tendencies for a need for a better introduction to, e.g., a handover between the primary and secondary healthcare sector were found in Flink et al.’s qualitative interview study. The study found that patients participated more actively in information handovers when they felt a need for involvement to ensure continuity of care, and were less active when they perceived that their contribution was unnecessary or not valued [27]. This highlights the need for a proper introduction to any written patient information to enhance a successful understanding.

Finally, the results from the present focus group interviews showed that the asthma patients were generally very positive about a leaflet with patient information about the new MART approach. They saw several advantages in implementing the leaflet in a conversation with their GP regarding a discussion of a switch to the new approach.

It is known that the meeting between patient and health care professional can be a difficult discipline, where the disclosure of important healthcare information, especially, can be a challenge. Research has, therefore, for a long time tried to examine what patients remember from a consultation with their GP, to identify possible solutions. In an exploratory pilot-study by Turner et al. from 2018, the introduction of a patient educational initiative prior to a deprescribing conversation between a healthcare provider and a patient changed the conversation from a monologue led by the healthcare provider to a more active conversation style with a higher proportion of dialogue [28]. An observational study by Richard et al. from 2017 showed that patients were able to better remember if they were activate in the conversation, and if the conversation had few and clear messages [29]. The authors also highlighted that the conversation must not be a monologue from the doctor, but rather a joint discussion between doctor and patient [29]. In relation to these findings, it could be suggested that a written patient-centered information leaflet could aid asthma patients when they had to discuss treatment changes with their GP. A review by Stacey et al. assessing the effects of decision aids in people facing treatment or screening decisions found that people exposed to decision aids felt more knowledgeable, better informed, and clearer about their values, and they probably had a more active role in decision making and more accurate risk perceptions. Whether our written patient-centered information leaflet could influence similar factors should be further investigated.

## 4. Materials and Methods

### 4.1. Design

This study was performed as a focus group study, which firstly examined a group of Danish asthma patients’ preferences, needs and attitudes towards printed patient information in relation to decision-making when changing treatment, and then used this information for the co-creation of a patient information leaflet. The present study is an elaboration of our previous study, also investigating factors influencing a successful switch to a new asthma treatment approach [23].

The case study involved a qualitative, semi-structured focus group interview method inspired by the theoretical framework for supporting patient involvement in research [17]. Focus groups were chosen over individual interviews to build collaboration and foster brainstorming, as interviewees listened to the views of others, motivating them to contribute with additional thoughts and ideas. 

The project was performed in a collaboration between the Department of Clinical Pharmacology at Copenhagen University Hospital Bispebjerg, the Faculty of Health and Medical Sciences at the University of Copenhagen and the patient organization Asthma-Allergy Association Denmark. The focus group interviews were performed at Copenhagen University Hospital Bispebjerg together with representatives from the patient organization Asthma-Allergy Association Denmark.

The study design adhered to the JBI Critical Appraisal Checklist for Qualitative Research [30] and the Consolidated Criteria for Reporting Qualitative Research (COREQ) [31].

### 4.2. Qualitative Focus Group Interview Method 

For asthma patients to co-develop an information leaflet about treatment change, qualitative semi-structured focus group interviews were used to explore asthma patients’ preferences and opinions about written patient-centered information. Two focus group interviews were held, with two slightly different foci:

1. Focus group:
  Focus on patients’ attitudes to information content and implementation before preparing the prototype information leaflet. The information leaflet prototype was developed based on the results of this focus group interview.
2. Focus group:
  Same focus, but the informants were asked to evaluate the information leaflet prototype and provide input for improvements.

After conducting the two focus group interviews, the content of the final information leaflet was determined based on input from both focus groups. 

The drafting of a semi-structured focus group interview guide was inspired by the theoretical framework for supporting patient involvement in research to ensure patient involvement in several study steps, including the domain of: identifying and prioritizing important study topics, informing the creation and reviewing proposed design, as well as giving feedback on implementation possibilities [17]. These steps were included in the semi-structured interview guide used in both focus group 1 and 2:

- Identification: asthma patients’ suggestions for the most relevant information that a written patient-orientated material should contain.
- Feedback on design: asthma patients’ preferences regarding a written patient-orientated leaflet—including the layout, language, and relevance of information.
- Implementation: the implementation of written patient-orientated material in the meeting with healthcare professionals such as staff at local community pharmacies and general practitioners, and how the material could be used in these meetings from the patients’ perspective.

Purposive sampling was used to recruit interviewees through a membership newsletter and an online registration form presented on the website of the patient organization Asthma-Allergy Association Denmark. In the online registration form, a series of personal questions regarding gender, age, education, asthma severity and socioeconomic status were asked so that the two focus groups could be arranged containing as heterogenic and representative a group as possible. 

We did not invite participants from the first focus group to participate in the second, as we did not want bias from the first experience to influence the second. We aimed for six informants of each focus group. Both focus group interviews were conducted by the pharmacist (SSH), who had experience with conducting qualitative research. The pharmacist acted as a moderator of the two focus group interviews to secure appropriate coherence with study focus and sufficient participation by all interviewees [32]. Besides this, the pharmacists did not actively participate in the interview. 

The semi structured interview guide used in both focus groups covered:

1. An introductory assignment where the interviewees could write down their immediate thoughts before consensus was reached in the group.
2. A thorough review of their pharmacological asthma treatment made by a clinical pharmacist, focusing on potential pharmacological treatment changes based on the new MART approach.
3. A brainstorm exercise:
  ○ In focus group one: suggestions for relevant information for an information leaflet and a ranking of the information based on importance.
  ○ In focus group two: suggestions for changes to the first prototype of the leaflet and a ranking of the changes based on importance.
  ○ A group discussion of the use of a patient-centered information leaflet in two different cases; at the local community pharmacy and at the general practitioner’s office, identified in our previous study [23].

Steps 1 and 3 were adapted to the specific focus group. The whole interview guide was pilot tested on a third person. Written informed consent was requested from all participants to participate. 

All focus group interviews were audio recorded, and field notes were made during and immediately after the focus group interviews and transcribed verbatim.

Transcripts were analyzed using systematic thematization [33]. The data were manually and independently coded and analyzed by the pharmacist (SSH) and a second researcher (NEK) to ensure consistency and reliability. Thematic coding analysis was used to analyze the focus group interview data [32]. Themes were derived directly from the interview data, and were not identified in advance. Through the coding process, if discrepancy over appropriate themes equivalent to specific codes arose, the two researchers discussed the disagreements until consensus was reached. All interviewees were adequately represented, and examples of quotes that emphasized the attitudes of the interviewees were highlighted. This process adhered to the Consolidated Criteria for Reporting Qualitative Research (COREQ) [31].

## 5. Limitations

The results from the focus group interviews were based on an online recruitment strategy at a patient organization webpage, which could have influenced the population sample. The asthma patients repeated during the interview that they may not have been the most representative group, as they were very knowledgeable about their disease and treatment. Therefore, the recruitment strategy may have influenced the results and future studies should seek to optimize this. However, attempts were made to minimize this bias by including as heterogeneous group of asthma patients as possible i.e., different disease severity, age, and sex.

The interview was limited by the focus group structure, where dominant interviewees may suppress less outspoken interviewees from arguing their case, although efforts were made to diminish this bias by the pharmacist leading the interview and securing speaking time for all interviewees. Overall, the focus group interview method was found beneficial as it opened a discussion and idea exchange by brainstorming and teamwork.

The first and second focus group interviews had slightly different foci, cf. Materials and Methods. It is our belief that the two foci supported the elucidation of the purpose of the study and that, despite the minimal difference between the foci, data saturation after two rounds of interviews was achieved. Thus, both focus group interviews identified similar important topics and similar attitudes towards appropriate implementation strategies [34].

## 6. Conclusions

In conclusion, the present study uniquely identified asthma patients’ preferences when co-developing written patient-centered information and assessing how the patients favored the material to be implemented as a support for them in their decision on whether to change asthma treatment. The results showed that asthma patients contributed with identifying relevant topics, and gave feedback on leaflet design and content as well as preferred implementation strategies. The included patients preferred written patient-centered material to be short and to be presented briefly at the local community pharmacy, and then discussed more thoroughly with their GP at a consultation meeting.

The asthma patients clearly stated that information about the effect, side-effects and use of the new MART approach was most important to them in relation to decisions about treatment change, and were key factors influencing their willingness to adhere to new treatment guidelines. A patient-centered information leaflet covering these important patient-reported topics may influence a more adherent asthma treatment. However, more research on this is needed to complete the information obtained from the present patients regarding compliance. Hence, additional patient data as well as interviews with pharmacists and GPs should be included in a future study.

This knowledge may be contributory in understanding comparable situations in other pharmacological settings.

## Figures and Tables

**Table 1:** A summary of the GINA Guidelines from 2018 compared with the GINA guidelines from 2022.

GINA Guideline Year	Step 1	Step 2	Step 3	Step 4
2018	SABA as needed	SABA as needed + low dose ICS	SABA as needed + low dose ICS/LABA combination	SABA as needed + medium dose ICS/LABA combination
2022	Low dose ICS + formoterol combination as needed	Low dose ICS + formoterol combination as needed	Low dose ICS + formoterol combination daily and as needed	Medium dose ICS + formoterol combination daily and SABA as needed

**Table 2:** Summary of patient characteristics of interviewees from both focus group interviews. * Information gathered from the online application form.

First Focus Group Interview				
Interviewee Abbreviation	Sex	Age	Severity of Asthma	Education *
F1A	Female	66	Mild	Long
F1B	Male	58	Severe	Long
F1C	Female	31–50 *	Severe	Long
F1D	Male	55	Severe	Vocational
F1E	Female	Over 71 *	Moderate	Vocational
Second Focus Group Interview				
Interviewee abbreviation	Sex	Age	Severity of asthma	Education *
F2A	Female	28	Severe	Long
F2B	Female	57	Mild	Vocational
F2C	Male	55	Severe	Vocational
F2D	Male	73	Severe	Vocational

**Table 3:** Themes, subthemes, and examples of quotes from both first and second focus group interviews.

Themes	Subthemes	Quotes
Identification of important topics about the new MART approach	Effect and side effects	“It’s a really big step to say goodbye to something that you know works, to something that some others say works, but that I’m not actually sure about.” (F1D)“After all, you get exactly the same side effects [with the new MART approach] as you would always get [with your current treatment].” (F2A)
	Usage	“When should I take it [the medicine]? And when do I have to see the doctor? When should I react to the fact that this is not controlled well enough?” (F1D)“But I think that if you were schooled in [taking the medicine as needed] from the start […]. In other words, we probably need to educate people more.” (F2A)
	Finance and access	“Is it [the new asthma medicine] something they have in stock at the pharmacy?” (F1C)“And then when you say those magic words: “It’s a little bit cheaper”—then you’re always up for sale.” (F2C)
	Symptom assessment	“As a new person with asthma [...] I would be very doubtful: “Is this asthma or is it something else?” Do I now have to use it [the medication]?”” (F1A)“I think I would end up using it [the inhaler] too little [if it only were as needed] Because I would tend to think: “Ah, it is not quite bad enough for me to take it”. Whereas the fixed routine of having to take it [the medicine] morning and evening makes me take it.” (F1D)“I think that if you don’t know your own illness very well, you might sometimes be a bit in doubt: “Should I take it [the inhaler] or shouldn’t I take it?” (F2C)
Feedback on the design of written patient-centered information	Format	“Maybe it [the leaflet] is almost a postcard size you get, where the names [of the medicine] are just there, so you can remember. And quite briefly.” (F1B)“I think there is a bit too much text […] and it is some difficult and long words to read, if you are not such a good reader.” (F2B)“F2D: And then it [the leaflet] must be much larger. It must be a real one that you can flip through. F2B: Yes, a booklet with some pictures.” (F2D and F2B)
	Better recollection	“It would be positive to have something in writing, because otherwise I would have forgotten it 26 s later.” (F1D)
	Increased patient autonomy	“But then you [with the leaflet] could say [to the doctor]: “Hello, it’s not just me who thinks this [new MART approach is advantageous], it’s also the Danish Pulmonary Society who thinks this and the Danish Capital Region.” (F2A)
	Information overload	“Basically, I don’t really believe in such leaflets here to be honest. Because today we are overwhelmed with information about all sorts of things.” (F2C)
Implementation of written patient-centered information	At the GP clinic	“It would be there [at the GP] that I would seek my primary advice on whether something needs to be changed in my medication and such.” (F1C)“I think that people are very loyal to the fact that they have to actually go to their GP with this problem.” (F2C)“It [the leaflet] is not something you just have handed out. There must be words along the way.” (F2D)
	At the pharmacy	“No, for me the pharmacy is a commercial business that just happens to have some legal rights to sell some goods that others are not allowed to sell.” (F1D)“I think it’s a good idea to be made aware: “There is something new [the MART approach]. You can just talk to your doctor about it.” I think that’s fine.” (F1E)“I think it depends on whether you trust the person you are talking to. It is very good [...] when the pharmacy asks if you know how to take your medicine. But sometimes I’m like: “I think you are entering an area where it is a relationship between patient and doctor”.” (F2C)“I think it’s a good idea that when a pharmacist dispenses asthma medicine, they can draw attention to this leaflet and say: “I would recommend that you talk to your doctor about it.” (F2B)

## Data Availability

Data is contained within the article.
